# Cytokine-Induced Killer Cells As Pharmacological Tools for Cancer Immunotherapy

**DOI:** 10.3389/fimmu.2017.00774

**Published:** 2017-07-06

**Authors:** Xingchun Gao, Yajing Mi, Na Guo, Hao Xu, Lixian Xu, Xingchun Gou, Weilin Jin

**Affiliations:** ^1^Shaanxi Key Laboratory of Ischemic Cardiovascular Disease, Institute of Basic Medical Sciences, Xi’an Medical University, Xi’an, China; ^2^State Key Laboratory of Military Stomatology, National Clinical Research Center for Oral Diseases, Shaanxi Engineering Research Center for Dental Materials and Advanced Manufacture, Department of Anesthesiology, School of Stomatology, The Fourth Military Medical University, Xi’an, China; ^3^Department of Instrument Science and Engineering, Institute of Nano Biomedicine and Engineering, Key Lab for Thin Film and Microfabrication Technology of Ministry of Education, School of Electronic Information and Electronic Engineering, Shanghai Jiao Tong University, Shanghai, China; ^4^National Centers for Translational Medicine, Shanghai Jiao Tong University, Shanghai, China

**Keywords:** cytokine-induced killer cells, immunotherapy, pharmacological tools, cancer therapy, clinical trials

## Abstract

Cytokine-induced killer (CIK) cells are a heterogeneous population of effector CD3^+^CD56^+^ natural killer T cells, which can be easily expanded *in vitro* from peripheral blood mononuclear cells. CIK cells work as pharmacological tools for cancer immunotherapy as they exhibit MHC-unrestricted, safe, and effective antitumor activity. Much effort has been made to improve CIK cells cytotoxicity and treatments of CIK cells combined with other antitumor therapies are applied. This review summarizes some strategies, including the combination of CIK with additional cytokines, dendritic cells, check point inhibitors, antibodies, chemotherapeutic agents, nanomedicines, and engineering CIK cells with a chimeric antigen receptor. Furthermore, we briefly sum up the clinical trials on CIK cells and compare the effect of clinical CIK therapy with other immunotherapies. Finally, further research is needed to clarify the pharmacological mechanism of CIK and provide evidence to formulate uniform culturing criteria for CIK expansion.

## Introduction

**Figure 1:**
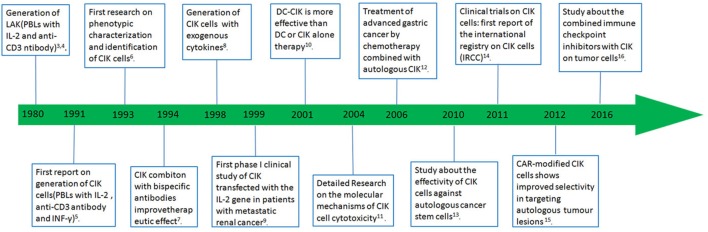
Selected highlights in the development of immunotherapy.

Cancer is among the top killer diseases and has emerged as a major public health problem around the world, and it will still be the main cause of the morbidity and mortality during the next few decades (1). Bray et al. estimated that the incidence of all cancer cases would up to 22.2 million in 2030 (2). In order to cure cancer, researchers have tried many antitumor strategies, but the recurrence and mortality rate of cancer are still high. Adoptive immunotherapy, as an adjuvant or alternative treatment, holds great promise in treating various malignant tumors. Cytokine-induced killer (CIK) cells are considered to be an ideal candidate cell type for cancer immunotherapy. A lot of basic researches and clinical studies show the safety and feasibility of CIK therapy in treating malignant tumors. Combination of CIK with either immunological or genetic engineering approaches have been made to improve the effects of CIK and all the key events in the history of CIK immunotherapy are shown in Figure 1 (3–16). In this review, the research progress and clinical application of CIK are summarized.

## What is CIK?

Cytokine-induced killer cells were first discovered in 1991 (5) and are a heterogeneous population of CD8^+^ T cells, which were generated from human peripheral blood lymphocytes (PBLs) and simply expanded *ex vivo via* incubation with an anti-CD3 antibody, interferon-γ (IFN-γ), and interleukin (IL)-2. They can kill tumor cells mediated by FasL and perforin (17). According to the presence of cell surface molecule CD56, CIK cells are also divided into two main subsets: CD3^+^CD56^+^ T cells and CD3^+^CD56^−^ T cells (18). CD3^+^CD56^+^ T cells, which are also called the natural killer T cells, are considered to be the major effector cells of CIK. So, CIK cells can lyse cancer cells in a MHC-unrestricted manner through activating NK cell receptors such as DNAX accessory molecule-1, NKp46, NKG2D, and NKp30 (11, 19, 20). In addition to the direct killing effect of CIK on cancer cells, they can also regulate the immune function by secreting various cytokines. A lot of studies have indicated that after stimulation by tumor cells, the levels of pro-inflammatory cytokines such as tumor necrosis factor (TNF)-α, IFN-γ, and IL-2 secreted by CIK cells are significantly upregulated (21), and these cytokines further enhance systemic antitumor activity and induce a Th1 immune response.

## *Ex Vivo* Expansion and Alloreactivity of CIK Cells

**Figure 2:**
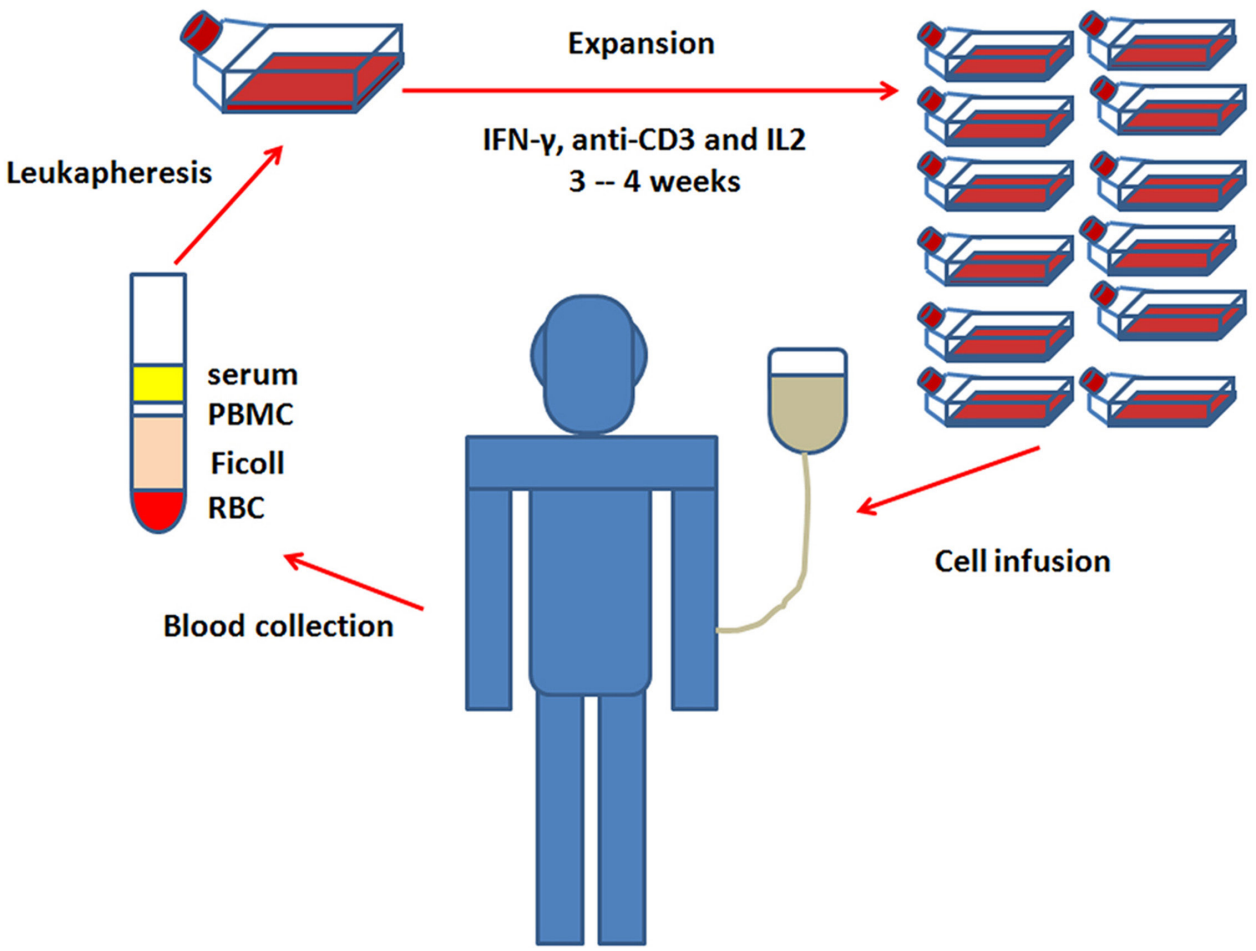
*Ex vivo* expansion of cytokine-induced killer cells and infusion.

Obtaining a sufficient number of antitumor immune cells is a critical step in the successful application of CIK cell immunotherapy (22). Fortunately, CIK cells can be easily expanded *in vitro* from peripheral blood mononuclear cells (PBMC), and some reports also showed that they could be also generated from umbilical cord blood precursors or bone marrow (23, 24). The general culture protocol for the *ex vivo* expansion of CIK cells requires 3–4 weeks with the addition of IFN-γ, anti-CD3 antibody, and IL-2. And the detail steps are as follows: on day 0, the PBMC are separated by density-gradient centrifugation from the whole blood (24, 25) and treated with IFN-γ to activate macrophages, which further provide cytokine-mediated (IL-12) and contact-dependent (CD58/LFA-3) signals to promote the cytotoxic power of CIK cells (26–28). On day 1, anti-CD3 antibody and IL-2 are added to the medium. Anti-CD3 will provide mitogenic signals for T cells which are then sustained by the continuous presence of IL-2 (29, 30). Fresh medium with IL-2 is added every 2 days. After 3–4 weeks of culture, the generated CIK cells are subsequently infused back into patients (Figure 2). The amount of injected CIK cells varied in different studies, so did the cell expansion rates. In fact, the average final expansion rates were usually in a range of 100-fold, but individual expansion rate was described to be variable from few to more than 1,000-fold (5, 25, 31, 32). It is well known that the more the CIK cells are injected and expanded, the better they response. Hontscha et al. showed that the total number of injected CIK cells ranged from 21.9 × 10^7^ to 5.2 × 10^10^ (14), Li et al. found the total number of CIK cells ranged from 6 × 10^6^ to 1.5 × 10^10^ in Chinese clinical trials (33). Until now, the least injected number of CIK cells was reported to be 6 × 10^6^ to obtain an objective response. Cohen et al. considered that tumor-infiltrating lymphocytes (TILs) must be expanded to 10^10^ for a successful treatment (34). Therefore, ~10^10^ CIK cells might be a good choice and many studies used more than 1 × 10^10^ cells to transfuse into the patients. As mentioned above, the reason why CIK expansion rate varies greatly is unclear. But there are still some additional strategies under investigation to further improve the expansion of CIK cells (22), which include adding new cytokines to the culture medium, such as IL-1, IL-7, IL-15, or thymoglobulin (8, 35, 36).

Cytokine-induced killer immunotherapy, a personalized therapy that uses patients’ own PBMC to expand antitumor CIK cells which are then reinjected into patients themselves, rarely causes autoimmune response. But sometimes, it is very difficult to obtain a sufficient number of CIK cells due to the poor health situation of patients, such as elderly people and patients with immunodeficiency diseases (37). To solve this problem, getting CIK cells from donor PBMC seems to be an alternative option. Studies showed that CIK cells exhibited a decreased alloreactivity across HLA barriers that could further reduce the risk of graft-versus-host disease (GVHD). Many phase I clinical studies proved that infusion of the allogeneic CIK cells in patients relapsing after allogeneic hematopoietic cell transplant would reduce the incidence of GVHD events (38–40). Another solution to obtain sufficient CIK cells is collecting from the cord blood. Mature protocols have already been made for generation of cord blood-derived CIK (CB-CIK) cells (41). The CB-CIK cells displayed relatively lower expression of HLA, indicating a weaker immunogenicity and lower risk of GVHD (42). Many clinical trials proved that CB-CIK cells were effective and safe to patients with malignancies (43, 44). All these suggest that CIK is a safe immune therapy with lower risk of GVHD.

## Improved CIK Therapy

**Figure 3:**
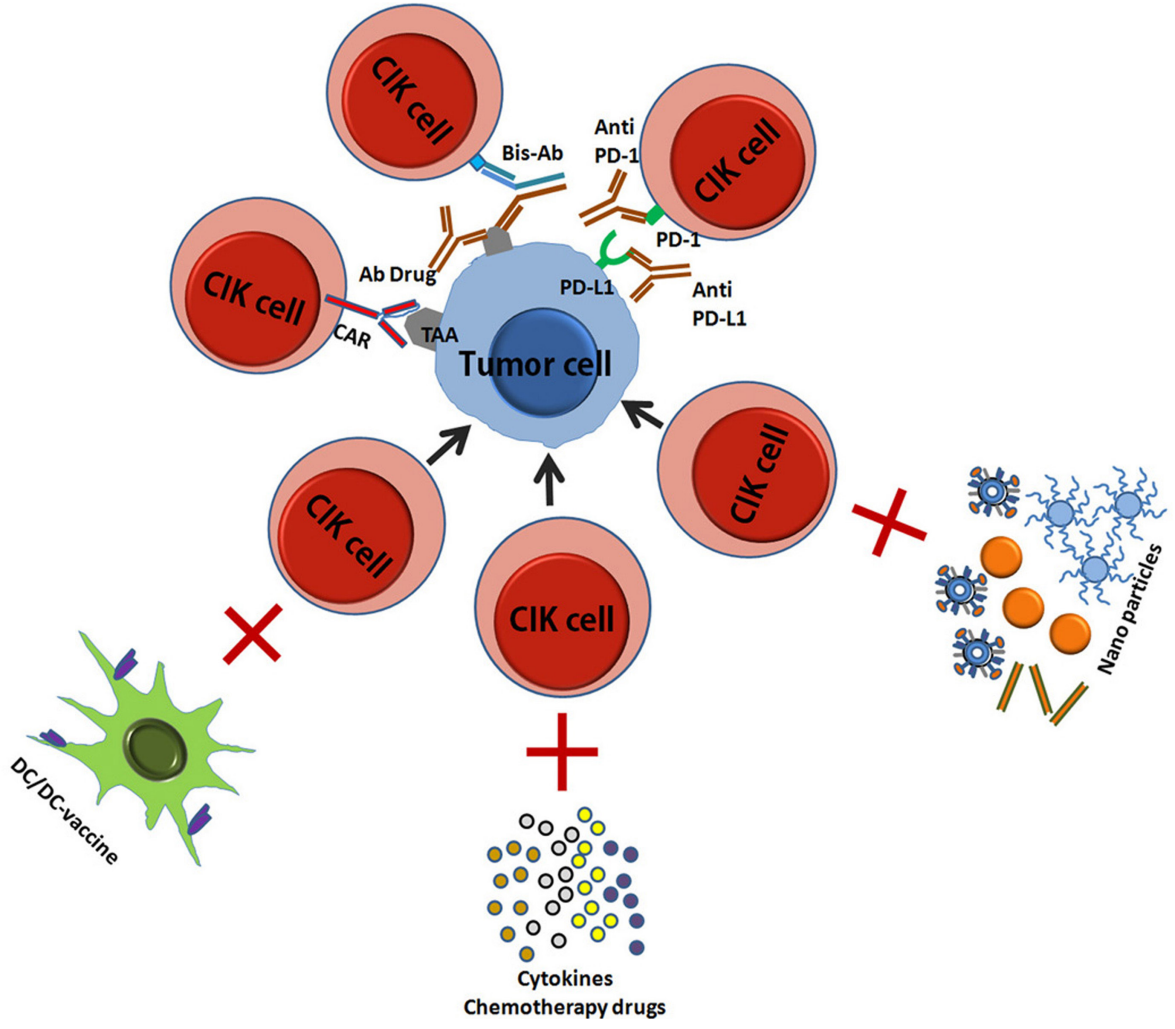
A schematic picture showed the improved CIK therapy. CIK, cytokine-induced killer; TA, tumor antigen; CAR, chimeric antigen receptor; Bis-Ab, bispecific antibody; Ab, antibody; DC, dendritic cells.

In the last decade, CIK cells have begun to be used in clinical care with good prospects for treatment success, and a great deal of research has been done to improve their cytotoxicity and safety. Here, we have summarized the current improved CIK therapies (Figure 3) (45, 46).

### CIK Combined with Additional Cytokines

Cytokine-induced killer cells are a heterogeneous cell population that can be expanded *ex vivo* from PBMC with the addition of IFN-γ, anti-CD3 antibody, and IL-2. In fact, many additional cytokines have been made to improve CIK antitumor activity. These cytokines can improve cell proliferation and cytotoxicity partly by suppressing the generation of regulatory T (Treg) cells that are known to inhibit antitumor immunity.

Lu et al. reported a new protocol for expansion of highly efficient cytotoxic CIK cells by culturing PBLs with addition of IL-1α (23). Another research showed that addition of IL-6 could significantly decrease the percentage of Treg cells and simultaneously increase the proliferation ability and cytotoxicity of the CIK cells against hepatocellular carcinoma (HCC) *in vitro* (47). By transfecting IL-7 gene expression vector into CIK cells, Finke et al. demonstrated an improved proliferation rate and enhanced antitumor cytotoxicity of CIK cells (48). Further study showed an upregulation of LFA-1 (CD11a/CD18) and CD28 in CIK cells with the addition of exogenous IL-7, which were essential for cytotoxic activity of CIK and played an important costimulatory role in T cell activation, respectively (49).

As an immune stimulatory cytokine, IL-12 has the strongest antitumor activity that can induce a Thl type of response and active NK and cytotoxic T lymphocyte (CTL) cells (50). CIK cells, generated with exogenous IL-12 instead of IL-2, showed similar cytotoxicity (8). However, due to its high toxicity, a lower dose of IL-12 should be used in combination with CIK in clinical application (50). Many studies demonstrated that CIK cells stimulated with IL-15 displayed improved proliferation capacity and cell cytotoxicity against hematologic and solid malignancies (36, 51–55). And a possible mechanism for its function might be the regulation of Treg cells and the expression of toll-like receptor 4, Wnt 4, PDGFD, and IL-35 (51, 56, 57). Application of IL-21 did not increase the proliferation rate of CIK cells (58, 59), but the cell cytotoxicity was significantly enhanced by increasing expression of IFN-γ, TNF-α, perforin, and granzyme B.

### Dendritic Cell (DC)–CIK

Dendritic cells are professional antigen-presenting cells (APCs) that can capture and process tumor-associated antigens (TAAs) (60). Given their particular ability to stimulate both adaptive and innate antitumor immune responses, DCs have been used as a powerful pharmacological tool for cancer immunotherapy (61). Many researches showed that DCs could promote NK cell-dependent antitumor effects through a cell-to-cell contact (62, 63). In recent years, studies have focused on the combinational treatment of DCs and CIK cells and proved a relative more safe and effective therapeutic effect on advanced solid carcinoma, which provide a new and efficacious immunity therapeutic strategy for cancer treatment. Cao et al. showed that coculture of CIK cells with DCs *in vitro* could improve the proliferation rate and antitumor activity of CIK cells (64). Further studies showed that the cellular interactions between CIK cells and DCs led to changes in the surface molecule expression of both populations and a significant increase of IL-12 secretion, and finally resulted in a higher cytotoxic activity of CIK cells (10). Pan et al. reported that DCs decreased the concomitant expanded Tregs in CIK cells and enhanced the cytotoxicity of CIK cells against leukemia cells (65). As we all know, delivering TAAs to DCs as vaccines have been reported to be an effective strategy for the treatment of various advanced malignancies. The combination of DCs vaccination with CIK cells is considered to be more prospected, and studies showed a significantly stronger antitumor activity and fewer side effects (17, 66–69). Jung et al. showed that in an *in vivo* animal model, the CIK + DC vaccination therapy was more effective than CIK or DC vaccination alone therapy for the treatment of hepatocarcinoma tumor cells (70). More recently, Lin et al. reported that DC–CIK therapy could improve survival by reducing the risk of disease progression in stage IV breast cancer patients (71).

### CIK Combined with Immune Checkpoint Inhibitors

Immune checkpoints are molecules that can either turn up or turn down signals in immune system. When CTLs recognize and trigger tumor cell death by inducing apoptosis, various checkpoint pathways between APCs/tumor cells and T cells are activated to provide signals for T cell activation (72, 73). There are at least two signaling pathways regulating the activation or inhibition of the CTLs: the primary signal is the binding occurred between peptide–MHC which is presented by APCs and T cell receptors. The secondary signal is the costimulatory/coinhibitory signal that regulates T cell activation (74, 75). The CD28, OX40, CD58, CD40L, CD80, CD86, and CD137 are stimulators that can promote immune activation, whereas programmed death 1 (PD-1), cytotoxic lymphocyte-associated antigen 4 (CTLA-4), lymphocyte activation gene 3 (LAG-3), T cell immunoreceptor with Ig and ITIM domains, and T-cell immunoglobulin and mucin-domain containing-3 (TIM-3) are inhibitors that suppress immune activation. CTLA-4 and PD-1 involved in the T-cell immune evasion in many malignancies, thus they are always designed as targets for cancer immune therapies (16, 76). It has been demonstrated that the blockade of inhibitory receptors such as PD-1, KIR, TIM-3, and LAG-3 but not CTLA-4 on CIK cells can significantly increase their antitumor potency against hematological malignancies. However, the combination of inhibitors against two receptors showed no increased cytotoxicity compared to that of one alone (77). Dai et al. reported that the blockade of PD-L1/PD-1 augmented CIK cytotoxicity against gastric and colorectal cancer cells. Additionally, combined therapy of CIK with checkpoint inhibitors (PD-L1/PD-1 blockade) could inhibit tumor growth and prolong the survival in the murine model of gastric cancer compared to untreated mice (16). All these suggest that combination of CIK cells with checkpoint inhibitors will be a novel immunotherapy for cancer treatment.

### CIK Combined with Antibodies

Immunotherapy of tumors with specific antibodies has been established as one of the most successful therapeutic strategies in the last 20 years. The mechanisms of antibody-based tumor cell killing are as follows: (1) antibody can directly bind to the cell surface receptor, deliver drug or toxin to target tumor cells, induce cell apoptosis, and reduce proliferation; (2) antibody can kill tumor cells by immune-mediated killing mechanisms such as activating complement, antibody-dependent cellular cytotoxicity, and regulating T cell function; (3) antibody can regulate tumor angiogenesis by vasculature receptor antagonism or ligand trapping (78, 79). Many studies showed that CIK cells combined with antibody could improve their cytolytic activity. Pievani et al. reported that addition of anti-CD20 mAb rituximab or GA101 could significantly enhance cytotoxicity of CIK cells to B-cell lymphoma *in vitro* (20). Deng et al. further proved that the enhanced CIK cytotoxicity induced by anti-CD20 mAb was partly related to the increased expression of components of the MAPK/ERK and STAT signaling pathways (80). Esser et al. reported that the combinational treatment of CIK cells with anti-CD30 mAb Brentuximab Vedotin (SGN-35) achieved better efficacy in CD30^+^ lymphoma, and SGN-35 did not affect the function of CIK cells (81). Besides, bispecific antibodies (BsAbs) that have two different antigen-binding sites could improve adoptive immunotherapy effect by cross-linking CIK cells with malignant tumor cells (82). BsAb CD19 × CD5 (HD37 × T5.16) has been reported to increase the cytolytic activity of CIK cells against B-lymphoma cells (83). BsAbs against cancer antigen-125 and Her2 significantly enhanced the cytotoxicity of CIK cells in primary ovarian cancer in both *in vitro* and *in vivo* models (84). BsAb EGFR/CD3 could improve the cytotoxic activity of CIK cells toward gastric cancer cells (85). When combined with anti-CD3/anti-CD133 BsAb, CIK cells showed significantly stronger cytotoxicity to CD133^high^ pancreatic and hepatic cancer cells than that of CIK, CD3-CIK, or BsAb alone (86). Recently, Ma et al. reported that CIK cells armed with BsAb CD3 × EGFR (EGFRBi-Ab) to target EGFR-positive glioblastoma significantly increased CIK cells cytotoxic activity *in vitro* and inhibited the growth of glioblastoma tumors in glioblastoma xenograft mice (87). All these data suggest a better *in vitro* and *in vivo* antitumor effect of CIK cells combined with antibodies, which will definitely provide a novel useful method for the CIK cells.

### CIK Combined with Chimeric Antigen Receptor (CAR)

To promote target cell recognition and improve specific cytotoxicity of CIK, CIK cells are always engineered with a CAR that is targeted to specific antigen. CIK cells engineered with a CAR that was directly targeted against carcino-embryonic antigen (CEA) showed improved activation toward CEA^+^ colon carcinoma cells, compared to that of CEA^−^ cells (15). Oelsner et al. proved that CD19 CAR-engineered CIK cells could dramatically enhance their antileukemic activity (88). Remarkably, Marin et al. found that CIK cells combined with CD19-target CARs containing a costimulatory CD28 or 4-1BB domain together with CD3ζ exhibited better antitumor effect than CARs based on DAP10 or CD3ζ alone (89, 90). Likewise, Hombach et al. reported that CIK cells arming with CD28-CD3ζ CAR showed stronger cytotoxicity; however, CIK cells arming with the third-generation CD28-CD3ζ-OX40 CAR that provided a super costimulation signal exhibited less antitumor efficacy due to increased activation-induced cell death (91). All these data indicate that CIK cells modified by CAR have stronger tumor cell killing activity, and the appropriate design for CAR is crucial for the successful application of CAR-mediated CIK response.

### CIK Combined with Chemotherapy

Many studies have shown that CIK/DC–CIK combined with different chemotherapy regimens for cancer treatment exhibited better efficacy than chemotherapy alone. Wu et al. showed that CIK cells plus chemotherapy (docetaxel and cisplatin) could significantly prolong the progression-free survival (PFS) and overall survival (OS) in advanced non-small-cell lung cancer (NSCLC) patients (92). Niu et al. proved that CB-CIK cells combined with second-line chemotherapy drug (dexamethasone) could improve PFS and OS in patients with advanced solid malignancies after first-line chemotherapy failure (44). As we all know, chemoresistance is a major problem to be solved in the treatment of cancers. Interestingly, many studies showed that the combination of CIK with chemotherapy could overcome chemotherapy resistance by activating the immune system (93, 94). Zheng and Han analyzed the efficiency and safety of chemotherapy combined DC with CIK cells in the treatment of NSCLC using meta-analysis. They found that chemotherapy combined with DC–CIK immunotherapy was superior in 1-year OS, disease control rate (DCR), and disease-free survival (DFS). Meanwhile, no more adverse events (AEs) appeared. However, there was no obvious improvement in objective response rate (ORR), partial response, 2-year PFS, and OS. All these suggested the combination therapy was modest in efficacy and safer for advanced NSCLC patients (95, 96). Similar results, with significantly prolonged 1–5 year OS, DFS, and improved DFS, ORR, and DCR with the infusion of CIK/DC–CIK combined with chemotherapy was reported by Mu et al. in patients with gastric cancer. Moreover, the levels of IFN-γ and IL-12, except IL-2, significantly increased after CIK/DC–CIK therapy, proving that the immune function of gastric cancer patients also significantly improved (97). Another meta-analysis proved that DC–CIK combined with chemotherapy could prolong 1-year OS, 2-year OS, and 3-year OS on colon cancer (98). All these data indicate an antitumor effect of CIK and chemotherapy in comparison with the use of chemodrugs or CIK cells alone.

### CIK Combined with Nanomedicine

With the rapid development of nanotechnology and nanomedicine, nanomaterials have been applied in various fields, especially in human health care. In recent years, nanotechnology has brought many new methods for cancer treatment (99–103). Nanometer-sized particles (1–100 nm) are in the same range of dimension as biomacromolecules including antibodies and membrane receptors (104). Their inherent physical/chemical properties or being loaded with different imaging/therapeutic agents in their surface, all of which make nanoparticles become a powerful tool for diagnosis, imaging, and therapy (105–107). Importantly, nanomaterials with low toxicity can be used as vaccine carrier/adjuvant to improve the immunogenicity of antigens by enhancing their cellular uptake, preventing enzyme degradation, and regulating the immune cells function (108, 109). Many researchers explored the potential applications of nanomaterials in monitoring the trafficking of DCs and enhancing the efficacy of DC-based cancer vaccines, such as quantum dots (110), magnetic nanoparticles (111–114), and upconversion nanoparticles (115). Furthermore, it has been reported that nanoparticles could also work as cancer vaccines (116), and these cancer nanovaccines could be envisioned as nanocarriers codelivering antigens and adjuvants (117–119). As we discussed, the efficacy of DC–CIK is better than DC or CIK alone, so we propose that the combination of CIK/DC–CIK cells with nanomaterials may have a great potential in cancer immunotherapy.

## Clinical Trials on CIK Cells

At present, CIK as a pharmacological tool for cancer therapy has been tested in clinical trials of various tumors. 90 registered clinical trials have been found on the website ClinicalTrials.gov (http://www.clinicaltrials.gov) (Table 1) by searching the keywords: cytokine-induced killer cells or CIK. One trial is working on psoriasis (Table 1). The majority of these are concentrated in China (58 studies), followed by 6 studies in U.S., 3 studies in Singapore, and 3 studies in Korea. Besides, two trials (NCT01533727, NCT02539017) have been withdrawn, 1 trial terminated (NCT01871480), 22 trials have been completed. Below, we will summarize these clinical trials on CIK cells.

**Table 1:** Clinical studies on cytokine-induced killer (CIK) cells.

Trial	Phases	Experimental design	Target disease	Recruitment	Enrollment
NCT01533727	II	CIK + chemotherapy	Non-small-cell lung cancer (NSCLC)	Withdrawn	0
NCT02539017	II	Dendritic cell (DC)/CIK + chemotherapy	Triple-negative breast neoplasms	Withdrawn	0
NCT01871480	II	CIK + gefitinib	NSCLC	Terminated	50
NCT01655628	II	CIK + chemotherapy	Nasopharyngeal carcinoma	Recruiting	40
NCT01902875	Undefined	CIK + chemotherapy	NSCLC	Recruiting	100
NCT01914263	I	CIK	Solid tumor	Recruiting	40
NCT01868490	I/II	CIK	Cholangiocarcinoma	Recruiting	13
NCT01186809	II	CIK	Hematologic malignancies	Recruiting	50
NCT01839539	II	DC–CIK	Colorectal cancer	Recruiting	60
NCT01799083	I/II	Decitabine + CIK	Solid tumors/B cell lymphoma	Recruiting	100
NCT00862303	I/II	DC–CIK	Renal cell carcinoma	Recruiting	100
NCT02621333	II	CIK + chemotherapy	Lung adenocarcinoma	Recruiting	280
NCT02280278	III	Radical surgery/adjuvant chemotherapy + CIK	Colon cancer	Recruiting	550
NCT01592422	II	CIK	Small-cell lung cancer	Recruiting	60
NCT01498055	II/III	CIK	Lung cancer	Recruiting	120
NCT01481259	II/III	CIK	NSCLC	Recruiting	120
NCT02752243	I/II	CIK	Myelodysplastic syndromes (MDSs)/acute leukemia	Recruiting	40
NCT02651441	I/II	DC–CIK + chemotherapy	NSCLC	Recruiting	60
NCT02568748	III	CIK	Advanced HCC	Recruiting	20
NCT02487017	II	DC–CIK + TACE	Hepatocellular carcinoma (HCC)	Recruiting	60
NCT02644863	II	DC–CIK + chemotherapy	Esophageal cancer	Recruiting	60
NCT01691625	Undefined	DC–CIK	Esophageal cancer	Recruiting	50
NCT01758679	IV	Licartin + CIK	HCC	Recruiting	120
NCT01783951	I/II	S-1 + DC–CIK	Gastric cancer	Recruiting	30
NCT01781520	I/II	S-1 + DC–CIK	Pancreatic cancer	Recruiting	30
NCT02504229	II	DC–CIK + chemotherapy	Gastric cancer	Recruiting	80
NCT01691664	Undefined	Radiation therapy + DC–CIK	Esophageal cancer	Recruiting	40
NCT01884168	Undefined	DC–CIK	Malignant tumor	Recruiting	30
NCT01898793	I	Chemotherapy + CIK	Leukemia, myeloid, acute	Recruiting	24
NCT01906632	Undefined	DC–CIK	Malignant tumor	Recruiting	50
NCT02851784	II/III	Microwave ablation + CIK	HCC	Recruiting	50
NCT01929499	II	CIK	Colonic neoplasms	Not yet recruiting	210
NCT01821495	II	DC–CIK to treat NPC	Nasopharyngeal carcinoma	Not yet recruiting	100
NCT02496988	IV	Temozolomide + CIK	Advanced milignant gliomas	Not yet recruiting	120
NCT02494804	I/II	Temozolomide + CIK	Milignant gliomas	Not yet recruiting	80
NCT02490735	II	CIK	Esophageal squamous cell carcinoma	Not yet recruiting	2,000
NCT01631357	II/III	CIK + chemotherapy	Lung cancer	Not yet recruiting	200
NCT01821482	II	DC–CIK	HCC	Not yet recruiting	100
NCT02497898	II	CIK	Lymphoma, non-Hodgkin	Not yet recruiting	1,000
NCT02487550	II	DC–CIK	Renal neoplasma	Not yet recruiting	1,200
NCT01235845	I/II	DC-activated CIK + DC	Malignant glioma	Not yet recruiting	30
NCT02415699	II/III	DC–CIK + chemotherapy	Colorectal cancer	Not yet recruiting	100
NCT01240005	I/II	DC–CIK	Renal cell carcinoma	Not yet recruiting	30
NCT01828008	Undefined	CD20 antibody + CIK	Lymphomas	Not yet recruiting	20
NCT02688686	I/II	Genetically modified DC + CIK	NSCLC with bone metastases	Not yet recruiting	30
NCT02498756	II	CIK + ipilimumab	Melanoma	Not yet recruiting	300
NCT02585908	I/II	CIK + γδ T	Gastric cancer	Not yet recruiting	120
NCT02856815	II	CIK	Hepatocellular	Not yet recruiting	78
NCT02782546	II	CIML NK cell	Acute myeloid leukemia	Not yet recruiting	60
NCT00769106	III	CIK	HCC	Completed	200
NCT02419677	II/III	Radiofrequency ablation + CIK	Colorectal cancer	Completed	60
NCT00815321	II	CIK	Chronic myeloid leukemia	Completed	11
NCT00394381	I/II	CIK	Acute myeloid leukemia/MDS	Completed	17
NCT01749865	III	CIK	Carcinoma, hepatocellular	Completed	200
NCT00460694	I/II	CIK	Hematological malignancies	Completed	24
NCT00477035	I/II	CIK	Hematologic malignancies	Completed	22
NCT01232062	Undefined	DC–CIK + chemotherapy	Triple-negative breast cancer	Completed	46
NCT02406846	Undefined	DC–CIK + cryosurgery	Neoplastic cells, circulating	Completed	80
NCT02412384	Undefined	DC–CIK + cryosurgery	Neoplastic cells, circulating	Completed	120
NCT02450448	Undefined	DC–CIK + cryosurgery	Neoplastic cells, circulating	Completed	60
NCT02416635	Undefined	DC–CIK + cryosurgery	Neoplastic cells, circulating	Completed	60
NCT02450357	Undefined	DC–CIK + cryosurgery	Neoplastic cells, circulating	Completed	60
NCT02450435	Undefined	DC–CIK + cryosurgery	Neoplastic cells, circulating	Completed	60
NCT02450422	Undefined	DC–CIK + cryosurgery	Neoplastic cells, circulating	Completed	60
NCT02425735	I/II	DC–CIK + γδ T	Liver cancer	Completed	40
NCT00807027	III	CIK + chemotherapy	Glioblastoma	Completed	180
NCT01395056	Undefined	DC–CIK + chemotherapy	Triple-negative breast cancer	Completed	23
NCT02425748	I/II	DC–CIK + γδ T	Lung cancer	Completed	40
NCT02418481	I/II	DC–CIK + γδ T	Breast cancer	Completed	40
NCT00699816	III	CIK	Hepatocelluar carcinoma	Completed	230
NCT00186342	Undefined	CIK	Hematologic malignancies	Completed	120
NCT02482454	II/III	Radiofrequency ablation + CIK	Cholangiocarcinoma	Active, not recruiting	50
NCT01392989	II	CIK + chemotherapy	MDSs	Active, not recruiting	44
NCT02489890	II	CIK	Urinary bladder neoplasms	Active, not recruiting	1,500
NCT00185757	I	CIK	Multiple myeloma	Active, not recruiting	20
NCT02485015	II	CIK + apatinib	Stomach neoplasms	Active, not recruiting	80
NCT02490748	II	Radiofrequency ablation + CIK	Cervical cancer	Active, not recruiting	10
NCT02493582	II	CIK + apatinib	Adenocarcinoma of lung	Active, not recruiting	400
NCT02215837	II	Chemotherapy + DC–CIK	Gastric cancer	Active, not recruiting	40
NCT01924156	I/II	adenovirus-transfected DC + CIK	Renal cell carcinoma	Active, not recruiting	30
NCT01898663	I/II	Adenovirus-transfected DC + CIK	High-risk soft tissue sarcoma	Active, not recruiting	30
NCT02491697	II	DC–CIK + capecitabine monotherapy	Breast cancer	Active, not recruiting	400
NCT02487693	II	Radiofrequency ablation + CIK	Ovarian carcinoma	Active, not recruiting	50
NCT02487992	II	CIK	Colorectal neoplasms	Active, not recruiting	1,200
NCT02693236	I/II	adenovirus-transfected autologous DC + CIK	Esophagus cancer	Active, not recruiting	30
NCT02202928	II	DC–CIK + chemotherapy	Colorectal cancer	Active, not recruiting	60
NCT01956630	I/II	DC–CIK	Acute leukemia	Active, not recruiting	25
NCT02688673	I/II	DC–CIK	Small-cell lung cancer	Active, not recruiting	30
NCT02678013	III	RFA + highly purified cytotoxic T lymphocytes	HCC	Active, not recruiting	210

These data were searched on 11 August, 2016 from the ClinicalTrials.gov (http://www.clinicaltrials.gov) using the word “cytokine-induced killer cells OR CIK.”

In a first Phase I study, Schmidt-Wolf et al. demonstrated the safety and initial activity of CIK in therapeutic trials (9). In addition, CIK cells could be successfully expanded from patients treated with or without chemotherapy (120), so CIK cells were widely applied to treat various types of tumors including HCC, lung cancer, and gastrointestinal tumors (Table 1).

Lee et al. reported that patients who received CIK immunotherapy after curative treatment for HCC had a 14-month median recurrence-free survival (RFS) benefit (121). The OS and cancer-specific survival were longer in the immunotherapy group compared to the control group. The ratio of AEs was significantly higher in the immunotherapy group (*P* = 0.002), but there was not a significant difference in the proportion of patients with serious AEs between groups (*P* = 0.15) (NCT00699816). It is worth noting that previous studies about CIK therapy for HCC showed significant benefits in preventing recurrence, but no significant survival gains (122–124). The difference of these results may be due to several aspects, such as the different intensified schedule of CIK, different cancer clinical stage or the non-standard quality of CIK cells (commercialized CIK cell compared to uncommercialized CIK cells). Lee et al. proved that adjuvant immunotherapy with activated CIK cells could increase the RFS and OS of patients who suffered with HCC (125) (NCT00699816). Furthermore, adjuvant CIK cells treatments were proved to be safe and effective for HCC treatment by several meta-analyses (126–129).

In 2012, a phase II clinical study showed that CIK immunotherapy could enhance the efficacy of conventional chemotherapy in patients with NSCLC (130). Furthermore, Chen et al. proved the MHC class I-related chain A (MICA) status was also associated with the outcome measures in CIK therapy for patients with gastric cancer, for the patients with high expression of MICA were more likely to benefit from CIK therapy (131, 132). In a phase II/III study, combined radiofrequency ablation with CIK has been reported to be a safe and effective treatment for CRLMs patients (133). Hereafter, many clinical studies were conducted to evaluate the efficacy of CIK/DC–CIK cell therapy for lung cancer, and all the results showed that CIK/DC–CIK was an effective therapy for lung cancer (134–139).

Many researches proved that CIK therapy was therapeutic for treatment of gastrointestinal tumors such as colorectal cancer and gastric cancer. In 2014, a meta-analysis in China showed that the combination of DC–CIK with chemotherapy could significantly improve the survival benefit, DFS rate, and overall response rate in patients with colon cancer. Furthermore, the number of CD4^+^ T cells was significantly increased in the DC–CIK + chemotherapy group (98). Zhao et al. reported that CIK cells could improve OS of metastatic colorectal cancer patients in a phase II clinical trial (140). Lin et al. showed that DC–CIK combined with chemotherapy could prolong PFS and OS in colorectal cancer patients compared to chemotherapy alone (141). Mu et al. summarized 17 eligible trials including 1,735 patients with gastric cancer in a meta-analysis, and they found that the combination of chemotherapy with CIK/DC–CIK significantly increased the OS rate and DFS rate, enhanced immune function, and reduced the AEs caused by chemotherapy (97). Kong et al. showed that the addition of CIK cells immunotherapy to standard chemoradiotherapy with temozolomide improved PFS but not OS in a phase III randomized trial of newly diagnosed glioblastoma in Korea (142) (NCT00807027).

The clinical outcome of cancer with different pathologic stages is different, but little is known about the achievable outcome of CIK cells in patients with different pathological stages of the tumor. Recently, Li et al. reported that combined CIK with conventional treatments could increase the survival rate of early-stage melanoma patients (143). So, whether the outcome of CIK is better in early-stage patient needs to be further studied.

As we have seen, most of the CIK registered clinical trials are restricted to Asian countries (64 trials), so it is difficult to completely evaluate the effects of CIK therapy over the world. Besides, the number of patients included in some clinical trials is also inadequate. So, more large-scale, grouped, controlled, multi-center, non-commercial clinical trials are required to confirm the immunotherapeutic effects of CIK in cancer treatment.

## Conclusion and Prospects

In recent years, immunotherapy is considered to be a powerful pharmacological tool for the treatment of many malignancies (144). It is known that the expression of tumor antigens and MHC-I molecules are often downregulated or completely lost on tumor cells. Although the TILs could recognize specific antigens expressed by autologous tumor cells, this specific antigenicity is too low to achieve a high degree of antigenicity in therapeutic use (120, 145). What is more, the extremely low numbers of TIL cells and lymphokine-activated killer (LAK) cells also restrict their application. Compared to LAK cells, the CIK cells exhibit a relative higher proliferation rate and stronger antitumor activity. Especially for their non-MHC-restricted characteristic, CIK cells have a much broader antitumor spectrum. Antibody-based therapy and CAR-T cell therapy for cancer have been demonstrated to be one of the most successful and important treatment strategies, but there are still some details to be improved. One of the key challenges is to identify suitable antigens of tumors with explicit target genes. Unfortunately, the presence of intrinsic or acquired resistance has blunted the advantages of targeted therapies, the reason is partially due to the mutation or downregulation of specific antigen (79). The immune checkpoint blockade shows impressive clinical results, but the majority of patients are either resistant or relapse after therapy (146). Based on these data, optimal adoptive cellular immunotherapy (ACI) may require a portfolio of different treatment strategies.

As mentioned earlier, CIK cells are a heterogeneous population of CD3^+^ T lymphocytes, which maybe not only the biggest drawback but also the biggest advantage of this method. As a heterogeneous cell population, CIK cells are proved to contain different subpopulations and present a mixed T–NK phenotype. Until now, the precise subpopulation that is the most crucial and relevant to the clinical outcome and the exact pharmacological mechanism of how CIK kill tumor cells are still not completely understood. So, it is hard to precisely evaluate the effect of the CIK cell on immune response in cancer patients and hard to know why some clinical trials with CIK failed. Clearly, this uncertainty limits the clinical application of CIK. In contrast, as a heterogeneous cell population, CIK cells are able to kill heterogeneous tumor cells despite the tumor heterogeneity and antigen escape, which are major setbacks for the antibody-based and CART immunotherapy (147). And this may be the biggest advantage of CIK therapy. CIK cells therapy can improve the prognosis of cancer patients for its safety and decreased recurrence, so it would be the most effective treatment on residual cancer cells after conventional therapy.

Recently, much effort has been made to improve the antitumor activity of CIK cells and the recent findings of clinical applicability are reviewed in the paper. The promising outcomes have been made by CIK cells therapy combined with other therapies. But further research is still needed to optimize the procedure of CIK therapy. First, uniform culturing criteria should be formulated for CIK expansion. Second, more clinical trials need to be conducted with large-scale, controlled, grouped patients, including patients with different tumor stages and different cancer biomarkers. Only by doing these, can we figure out the tumor-killing mechanism of CIK cells and better evaluate their clinical efficacy.

More and more people realize that it is hard to cure cancer with only one drug or one therapeutic strategy. The combination of different drugs or therapies has already become a trend in treating heterogeneous tumors. As an effective ACI, CIK cells therapy provide a chance to prolong survival of cancer patients in clinical practice, and it is definitely worthy to spend more effort on it.

## Author Contributions

XG, YM, and WJ conceived this study. XG and NG wrote the manuscript. HX and LX provided critical discussion in manuscript preparation. LX and WJ revised the manuscript. All the authors reviewed this manuscript.

## Conflict of Interest Statement

The authors declare that the research was conducted in the absence of any commercial or financial relationships that could be construed as a potential conflict of interest.
